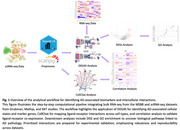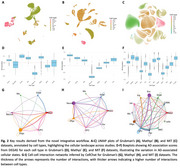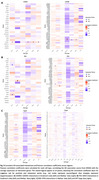# Unraveling High‐Risk Cell‐Cell Interactions in Alzheimer's Disease Using an Integrative Single Cell Analysis

**DOI:** 10.1002/alz70855_105077

**Published:** 2025-12-24

**Authors:** Jiahui Liu, Jie Zhang, Kun Huang, Travis S Johnson

**Affiliations:** ^1^ Indiana University, School of Medicine, Indianapolis, IN, USA; ^2^ Indiana University, Fairbanks School of Public Health, Indianapolis, IN, USA; ^3^ Indiana Biosciences Research Institute, Indianapolis, IN, USA

## Abstract

**Background:**

Alzheimer's disease (AD) is a complex neurodegenerative disorder characterized by cognitive decline and disruptions in neurovascular integrity, inflammation, immune signaling, and synaptic connectivity. While single‐cell and bulk RNA‐seq have revealed cell‐type‐specific changes in AD, the intercellular communication networks driving these processes remain unclear. This study introduces a novel integrative pipeline to systematically identify and prioritize robust cell‐cell interactions contributing to AD pathology.

**Method:**

We developed an advanced computational pipeline (Figure 1) that, to our knowledge, is the first to integrate cell prioritization tools with cell‐cell interactions to identify AD‐associated interactions. To achieve this, we integrated RNA‐seq from the Mount Sinai Brain Bank (MSBB) and single‐cell RNA‐seq (scRNA‐seq) from Grubman, Mathys, and MIT datasets, totaling over 2.4 million cells. The pipeline combined Diagnostic Evidence GAuge of Single cells (DEGAS), a transfer learning framework for identifying AD‐associated cellular states, with CellChat, which infers intercellular communication networks by mapping ligand‐receptor interactions across cell types. To ensure robustness, correlation analysis validated ligand‐receptor co‐expression, while differential gene expression (DEG) and gene ontology (GO) analyses identified pathways linked to AD‐associated states. Consistent interactions across datasets will be experimentally validated via colocalization using immunofluorescence (IF) and binding via co‐immunoprecipitation (Co‐IP).

**Result:**

Our pipeline consistently identified cell‐cell interactions underlying AD pathology across datasets (Figure 2). FN1‐SDC4 interactions between endothelial cells and astrocytes were associated with blood‐brain barrier dysfunction (Figure 3). CADM1‐CADM1 interactions, observed between oligodendrocytes and oligodendrocyte progenitor cells (Figure 3), highlighted their role in synaptic organization and connectivity deficits. The BSG‐PPIA interaction, specific to endothelial cells, was linked to vascular inflammation (Figure 3). Additional interactions, JAM3‐JAM3 and PTPRM‐PTPRM, were consistently identified in control contexts, suggesting roles in synaptic maintenance and axonal guidance. Correlation analysis further validated ligand‐receptor co‐expression, while DEG and GO analyses highlighted enriched pathways, including extracellular matrix organization, cell adhesion, and synaptic signaling. These results are now being further validated via IF and Co‐IP.

**Conclusion:**

This study highlights the novelty of our pipeline in integrating RNA‐seq data to identify robust intercellular communication networks in AD. By prioritizing consistent interactions across datasets, we provide insights into AD pathology that inform experimental validation and therapeutic development.